# Association of Ideal Cardiovascular Metrics and Serum High-Sensitivity C-Reactive Protein in Hypertensive Population

**DOI:** 10.1371/journal.pone.0081597

**Published:** 2013-12-11

**Authors:** Hao Xue, Jianli Wang, Jinhong Hou, Hang Zhu, Jingsheng Gao, Shuohua Chen, Yutang Wang, Yundai Chen, Shouling Wu

**Affiliations:** 1 Department of Cardiology, Chinese People’s Liberation Army General Hospital, Beijing, People’s Republic of China; 2 Department of Cardiology, Kailuan Hospital, Hebei United University, Tangshan, People’s Republic of China; The University of Manchester, United Kingdom

## Abstract

Increased levels of the inflammatory biomarker high-sensitivity C-reactive protein (hs-CRP) are associated with increased risk of cardiovascular disease. However, ideal cardiovascular health indicates lower risk of cardiovascular disease. This study aimed to investigate the effect of ideal cardiovascular health behaviors and health factors on hs-CRP levels among a hypertensive population. From 2006 to 2007, a cross-sectional study was conducted to survey 41,476 hypertensive subjects among the employees of Kailuan Corporation. Data from unified questionnaires and blood biochemical examinations were collected. The effects of ideal cardiovascular health behaviors and health factors on hs-CRP levels were evaluated through multivariate logistic regression analysis. A negative correlation was observed between hs-CRP levels and the number of ideal cardiovascular health metrics. The mean hs-CRP levels of subjects with zero to one, two, three, and four to six ideal cardiovascular health metrics were 1.11, 0.96, 0.90, and 0.80 mg/L, respectively (P<0.01). Multivariate logistic regression analysis revealed that after adjustment for sex, age, triglyceride, low-density lipoprotein cholesterol, high-density lipoprotein cholesterol, and other risk factors, the risks for subjects with two, three, and four to six ideal health metrics with serum hs-CRP >3 mg/L were lower than those with zero to one ideal health metrics, with corresponding odd ratios of 0.86 (95%CI: 0.79–0.93, P<0.01), 0.76 (95%CI: 0.69–0.83, P<0.01), and 0.68 (95%CI: 0.64–0.75, P<0.01), respectively. This finding suggests that ideal cardiovascular health behaviors and health factors were related to decreased hs-CRP levels in a hypertensive population.

**Clinical Trial Registration:**

Unique identifier: ChiCTR-TNC-11001489.

## Introduction

Cardiovascular disease (CVD) is the leading cause of death in China. Hypertension is the most important risk factor for CVD. According to recent epidemiological data, the rising prevalence of hypertension in China has reached approximately 200 million people, which means that one fifth of adults currently suffer from hypertension in China [1]. Numerous studies have confirmed that hypertension is closely connected to lifestyle, which is directly correlated with the risk of CVD. In 2010, the American Heart Association defined and quantified ideal cardiovascular health behaviors and health factors, and set a health strategic goal of “By 2020, to reduce 20% of the deaths from cardiac cerebrovascular diseases by improving Americans’cardiovascular health behavior factors” [2]. Studies showed that high-sensitivity C-reactive protein (hs-CRP) acts as an inflammatory marker, is associated with increasing cardiovascular risks, and plays an important role in hypertension and atherosclerosis [3], [4]. Furthermore, adverse cardiovascular health metrics and clinical factors, such as obesity [5], unhealthy diet [6], lack of physical activity [7], smoking, dyslipidemia, diabetes, and hypertension, are associated with higher hs-CRP levels [8], [9]. However, no scientific report currently exists regarding the association between the number of ideal cardiovascular health behaviors and factors and chronic inflammatory levels among hypertensive populations. Thus, this study aimed to investigate the effect of ideal cardiovascular health behaviors and health factors on hs-CRP levels among a hypertensive population in the Kailuan study.

## Materials and Methods

### Subjects

From July 2006 to October 2007, a total of 101,510 working and retired employees of the Kailuan Corporation underwent the first-time physical examinations at the Kailuan General Hospital and its 10 affiliated hospitals: Kailuan Lindsey Hospital, Kailuan Zhaogezhuang Hospital, Kailuan Tang Village Hospital, Kailuan Fangezhuang Hospital, Kailuan Lujiatuo Hospital, Kailuan Jinggezhuang Hospital, Kailuan Linnancang Hospital, Kailuan Qianjiaying Hospital, Kailuan Majiagou Hospital, and Kailuan Hospital Branch.

Blood pressure at the subject’s left upper arm in the sitting position was measured by professional doctors with a standardized mercury sphygmomanometer after at least 5 min rest. Two readings each of systolic blood pressure (SBP) and diastolic blood pressure (DBP) were taken at a 5-min interval. The average of the two readings was used for data analysis. Hypertension was defined according to the 7th edition report of the USA Joint National Committee on Prevention, Detection, Evaluation and Treatment of Hypertension [10]: as SBP ≥140 mmHg and/or DBP ≥90 mmHg on average of two measurements or by current antihypertensive treatment. A total of 44,653 hypertensive patients were recruited into this study. Patients with the following criteria were excluded: (1) incomplete data related to the study; (2) serum hs-CRP>10 mg/L (Previous study has shown that hs-CRP level of >10 mg/L often represents acute inflammations. So, subjects with hs-CRP level of >10 mg/L were excluded in our study) [11]; (3) infectious diseases, cancer, hematological diseases, severe liver disease, cardiac or renal failures, or autoimmune diseases; (4) secondary arterial hypertension; (5) immunomodulator use in the past 3 months; and (6) recent surgical and traumatic history. From the above, a total of 3,076 (6.9%) subjects were excluded from this study. A sum of 41,577 subjects was finally recruited as the study population. The cross-section study was conducted according to the guidelines of Helsinki Declaration and approved by the Ethics Committee of the Kailuan General Hospital. All participants signed informed consent forms. All participants were of Han nationality.

### Data Collection

A detailed medical history was obtained from all subjects by questionnaire assessment as previously described [12]–[13], including family history of hypertension, diabetes mellitus, coronary heart disease and stroke. The following conventional cardiovascular risk factors were also recorded: alcohol intake, smoking status, salt intake data, physical activity, and body mass index (BMI). BMI was calculated by using the formula of weight (kg)/height (m^2^).

### Biochemical Variables Determination

Blood samples were collected in tubes containing EDTA after an overnight fast, and were centrifuged at 3000 g for 10 min (centrifuge radius of 17 cm) at room temperature to isolate plasma. The supernatant serum was measured within 4 hours. Fasting blood glucose (FBG) was measured by hexokinase/glucose-6-phosphate dehydrogenase method. High-density lipoprotein cholesterol (HDL-C), low-density lipoprotein cholesterol (LDL-C), total cholesterol and triglyceride were measured enzymatically (inter-assay coefficient of variation <10%; Mind Bioengineering Co. Ltd, Shanghai, China). All biochemical variables were measured by using an automatic biochemical analyzer (Hitachi 747; Hitachi, Tokyo, Japan) at the central laboratory of the Kailuan General Hospital.

### Determination of Plasma hs-CRP Levels

The plasma hs-CRP concentrations were determined using a commercial, high-sensitivity particle-enhanced immunonephelometry assay (Cias Latex CRP-H, Kanto Chemical Co. Inc, Tokyo, Japan) with the detection limit of 0.1 mg/L. The intra-assay and inter-assay variability of hs-CRP were 6.53% and 4.78%, respectively.

### Ideal Health Cardiovascular Health Behaviors and Health Factors

The exercise definition was slightly different from the AHA definition (the ideal value defined by the AHA is ≥30 minutes each time, ≥5 times each week). Ideal cardiovascular health behaviors were defined as follows: (1) no-smoking; (2) body mass index (BMI) <25 kg/m^2^; (3) physical exercise: ≥30 minutes each time, ≥3 times each week; and (4) salt intake: low-salt diet (salt intake is less than 6 g/d). Ideal cardiovascular health factorswere defined as follows: (1) untreated total serum cholesterol <200 mg/dl, (2) untreated blood pressure: systolic pressure <120 mmHg and diastolic pressure <80 mmHg, and (3) untreated fasting blood glucose <6.1 mmol/L.

### Statistical Analysis

All statistical analysis was conducted by using SPSS 13.0. Continuous variables were described by means ± standard deviations and the comparisons between two groups were tested by t-tests or non-parametric tests based on distributional properties. Categorical variables were described by percentages and the comparison between two groups was conducted via chi-square (χ^2^) test. Hs-CRP level were presented as median (with interquartile range), because it was not normally distributed. Hs-CRP level of 3 mg/L was chosen as the threshold value. And, the hs-CRP>3 mg/L and hs-CRP≤3 mg/L were used as a dichotomous variable to analyze the effects of ideal cardiovascular health behaviors and health factors on plasma hs-CRP levels by using binary logistic regression. Two-sided P-values<0.05 was considered to be statistically significant.

## Results

### Characteristics of Hypertensive Patients

According to our previous study, patients were divided into two groups, namely, hs-CRP>3 mg/L group and hs-CRP≤3 mg/L group [10]. Compared with the hs-CRP>3 mg/L group, the hs-CRP≤3 mg/L group was generally younger and had significantly lower proportions of females to males, smoking history, and intake of lipid-lowering drugs, and higher proportions of ideal body mass index (BMI), ideal total cholesterol levels, ideal physical activity, and ideal fasting blood glucose (all *P*<0.01) (Table 1).

**Table 1:** The baseline Characteristics of the hypertensive population.

	hs-CRP≤3 mg/L	hs-CRP>3 mg/L	*F/χ* ^2^ Value	*P* Value
Gender Female (%)	4634 (13.8)	1508 (19.3)	151.41	<0.01
Age (Year)	54.7±11.3	58.6±11.1	−27.04	<0.01
Non-smoking n (%)	19794 (59.0)	13767 (41.0)	53.25	<0.01
Ideal BMI n (%)	10027 (29.8)	1994 (25.5)	58.12	<0.01
Ideal diet n (%)	3109 (9.3)	593 (8.4)	7.55	<0.01
Ideal physical activity n (%)	6372 (19)	1242 (17.6)	7.20	<0.01
Ideal TC n (%)	17566 (52.2)	3626 (46.3)	88.69	<0.01
Ideal blood glucose n (%)	26028 (77.4)	5253 (67.1)	262.20	<0.01
LDL-C (mmol/L)	2.50±0.84	2.20±1.32	19.37	<0.01
HDL-C (mmol/L)	1.57±0.41	1.56±0.43	1.57	>0.1
TG (mmol/L)	1.87±1.49	1.93±1.48	−3.25	<0.01
SBP (mmHg)	147.8±17.7	149.4±18.4	−6.76	<0.01
DBP (mmHg)	92.7±10.3	92.1±10.8	4.56	>0.1
Lipid-lowering drugs n (%)	518 (1.5)	166 (2.1)	24.07	<0.01
Drinking n (%)	19715 (58.7)	4582 (64.4)	78.88	<0.01

Note: Continuous variables were presented as mean ± standard deviation (SD), and dichotomous variables as numbers and percentages. BMI: Body Mass Index; FBG: Fasting Blood Glucose; TC: Total Cholesterol; TG: Triacylglycerols; HDL-C: High Density Lipoprotein Cholesterol; LDL-C: Low Density Lipoprotein Cholesterol; SBP: Systolic blood pressure; DBP: Diastolic blood pressure.

### Comparison of Serum hs-CRP Levels among Different Groups of Ideal Cardiovascular Health Metrics

**Figure 1:**
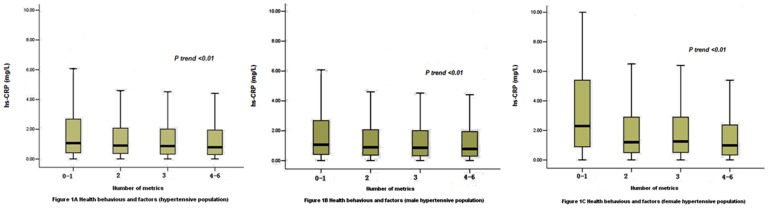
Serum hs-CRP indifferent groups of Ideal Cardiovascular Health Metrics in the hypertensive population, different genders hypertensive population. The hs-CRP levels are presented as box (median, 25th percentile, 75th percentile) and whisker (2.5th and 97.5th percentiles) plots.

Participants were divided into four groups according to the number of ideal cardiovascular health behaviors and health factors. Subjects with zero or one ideal health behaviors and health factors were classified as metrics 0 to 1, subjects with two ideal health behaviors and health factors were classified as metrics 2, subjects with three ideal health behaviors and health factors were classified as metrics 3, and subjects with four, five, or six ideal health behaviors and health factors were classified as metrics 4 to 6. The median hs-CRP levels the four groups as following: 1.11 (0.46–2.90) mg/L (0–1 metrics), 0.96 (0.40–2.20) mg/L (2 metrics), 0.90 (0.35–2.12) mg/L (3 metrics), and 0.80 (0.30–2.01) mg/L (4–6 metrics), respectively (P<0.01), which were negatively correlated to the number of ideal cardiovascular health metrics. Similar correlation was observed by gender, with the median hs-CRP levels 1.08 (0.42–2.70) mg/L (0–1 metrics), 0.90 (0.37–2.07) mg/L (2 metrics), 0.87 (0.33–2.01) mg/L (3 metrics) and 0.79 (0.30–1.95) mg/L (4–6 metrics) in male, and 2.30 (0.89–5.40) mg/L (0–1 metrics), 1.20 (0.50–2.90) mg/L (2 metrics), 1.25 (0.51–2.90) mg/L (3 metrics) and 0.99 (0.34–2.37) mg/L (4–6 metrics) in female (all P<0.01), respectively (Fig. 1A–1C).

### Effect of Ideal Cardiovascular Health Behaviors and Health Factors on Serum hs-CRP Levels

In logistic model, hs-CRP levels (≤3 mg/L = 0 and >3 mg/L = 1) as a dichotomous variable were defined as dependent variables. The numbers of ideal cardiovascular health behaviors and health factors were defined as independent variables (metrics 0 to 1, metrics 2, metrics 3, and metrics 4 to 6; metrics 0 to 1 was set as the control). Multivariate logistic regression analysis confirmed the aforementioned negative correlation that the risk of serum hs-CRP level of >3 mg/L remarkably declined with the increase in the number of ideal health metrics and health factors. After adjustment for gender, age, triglycerides, low-density lipoprotein cholesterol (LDL-C), high-density lipoprotein cholesterol (HDL-C), and other risk factors, the risks for subjects with two, three, and four to six ideal health metrics with serum hs-CRP >3 mg/L were lower than those in the control group (0–1 metrics), with corresponding odd ratios of 0.86 (95%CI: 0.79–0.93, P<0.01) (2 metrics), 0.76 (95%CI: 0.69–0.83, P<0.01) (3 metrics), and 0.68 (95%CI: 0.64–0.75, P<0.01 (4–6 metrics), respectively (Table 2).

**Table 2:** Impact of Ideal Cardiovascular Health Behaviors and Health Factors on hsCPR Levels.

No. of Metrics	Model 1*			Model 2*			Model 3*		
	OR	95%CI	P	OR	95%CI	P	OR	95%CI	P
0–1	1.00			1.00			1.00		
2	0.69	0.64–0.74	0.001	0.62	0.58–0.67	0.001	0.86	0.79–0.93	0.001
3	0.66	0.61–0.71	0.001	0.63	0.58–0.68	0.001	0.76	0.69–0.83	0.001
4–6	0.64	0.60–0.68	0.001	0.59	0.56–0.63	0.001	0.68	0.64–0.75	0.001

Model 1: Single-factor analysis; Model 2: Adjustment for gender and age; Model 3: Further adjustment for TG, LDL-C, HDL-C, and administration history of lipid-lowering drugs based on Model 2.

## Discussion

This study was the first to investigate the relations between ideal cardiovascular health behaviors and health factors and hs-CRP levels among hypertensive population. In this cross-sectional study, we found that the number of cardiovascular health behaviors and health factors was negatively associated with hs-CRP levels in hypertensive patients.

Previous population-based cross-sectional study confirmed that CRP levels gradually increase with increasing number of metabolic syndrome components (defined by the updated NCEP-ATPIII for Asian-Americans) [14]. This result was consistent with our finding that the number of ideal cardiovascular health metrics was negatively correlated with hs-CRP levels in hypertension. In addition, a large number of epidemiological and clinical studies have confirmed the negative correlation between the number of ideal health metrics and cardiovascular events [15]–[18]. Moreover, lifestyle intervention (such as exercise and healthy diet) or pharmacological treatments (such as intake of lipid-lowering agents) may be related to decreased hs-CRP levels [8]. These results show that more ideal cardiovascular health behaviors and factors associated with lower hs-CRP levels may translate into the prevention of CVD.

Hypertension is the most significant risk factor for CVD. Over 50% of cardiovascular mortality events are associated with hypertension [1]. Hs-CRP, as an inflammatory marker, is not only associated with increased risk of CVD, but also plays an important role in the incidence and progression of hypertension [19]. Every 1 mmHg increase in systolic blood pressure can result in a 3% increase in the risk of having hs-CRP levels ≥2 mg/L in the Inuit population [20]. The study on different genders of hypertensive patients demonstrated that the risk of CRP level being >3 mg/L is 1.4 times higher among females and 1.6 times higher among males than those in non-hypertensive patients [21]. The hs-CRP levels further increase if the hypertensive patients have morbidities, such as diabetes or metabolic syndrome [22].

The number of ideal cardiovascular health behaviors and health factors was also closely correlated with hs-CRP levels in a non-hypertensive population. The BMI increase of 0–0.6, 0.6–1.7, 1.7–2.8, and ≥2.8 kg/m^2^ every five years corresponded to risk increases of 2.27, 4.24, 5.88, and 11.8 times, respectively. The risk of hs-CRP of >3 mg/L increases with smoking frequency [23]. Weight loss, physical exercise, smoking cessation, and other lifestyle interventions could significantly reduce CRP levels and the risk of hs-CRP of >3 mg/L. Meanwhile, serum hs-CRP levels were closely related to blood glucose and lipids. The increase in CRP levels was also associated with elevated LDL-C and decreased HDL-C levels [8], as well as elevated fasting blood glucose levels [14].

The application of Dietary Approaches to Stop Hypertension can significantly reduce serum hs-CRP levels in patients with type2 diabetes [24]. The findings of our study were consistent with those of previous studies, in which the number of ideal cardiovascular health behaviors and health factors is negatively correlated with the serum hs-CRP levels in hypertensive populations. These results suggest that the cardiovascular protective effects of ideal cardiovascular health behaviors and factors could result from the reductions in serum hs-CRP levels through complex mechanisms, such as reductions in BMI, total cholesterol, and fasting blood glucose. The pathogenic mechanisms of the inverse relationship between ideal cardiovascular health factors and hs-CRP remain unclear. Previous studies showed that hyperglycemia and hyperlipidemia can cause endothelial dysfunction by affecting nitric oxide synthesis or degradation, which may increase hs-CRP levels [25]. In addition, smoking may induce systemic inflammation through an oxidative stress pathway. Physical activity may reduce systemic inflammation via increased insulin sensitivity. Previous research suggested that adipose tissues stimulate the secretion of interleukin-6, which is involved in the production of CRP in hepatocytes [26], [27]. All these findings may partly explain the negative association between ideal cardiovascular health factors and hs-CRP.

### Strengths/Limitations

The strength of this study was its large sample size and high participation rates during a two-year period. However, this study also had several limitations. First, salt intake was used to replace vegetable intake, and this modification was made based on the effect of salted food intake on CVDs in China and the recommendation on cardiovascular health standards by Chinese authorities. Second, the main source of research participants was the Kailuan Mine Corporation, with more males than females enrolled in the study cohort. The representativeness of the study was limited by the higher proportion of male subjects. Application of these results to other areas should be made with caution. Finally, as a cross-sectional survey, this study could not establish the causative correlation between ideal cardiovascular health behaviors and health factors and the change in serum hs-CRP levels among the subjects.

### Conclusions

Our findings indicate that increasing numbers of ideal cardiovascular health behaviors and health factors were associated with decreased hs-CRP levels. Thus, advocating ideal cardiovascular health behaviors may reduce hs-CRP levels and translate into the prevention of CVD.

## References

[1] Ministry of health of the people’s Republic of China (2010) China Health Statistics Yearbook [M]. Peking Union Medical College press. 276: 315.

[2] Lloyd-Jones DM, Hong Y, Labarthe D, Mozaffarian D, Appel LJ, et al (2010) Defining and setting national goal for cardiovascular health promotion and disease reduction: The American heart association’s strategic impact gola through 2020 and beyond. Circulation 121: 586–613. 20089546 10.1161/CIRCULATIONAHA.109.192703

[3] Higashiyama A, Wakabayashi I, Kubota Y, Adachi Y, Hayashibe A, et al (2012) Does high-sensitivity C-reactive protein or low-density lipoprotein cholesterol show a stronger relationship with the cardio-ankle vascular index in healthy community dwellers?: the KOBE study. J Atheroscler Thromb 19: 1027–34. 22785137 10.5551/jat.13599

[4] Shafi Dar M, Pandith AA, Sameer AS, Sultan M, Yousuf A, et al (2010) hs-CRP: A potential marker for hypertension in Kashmiri population. Indian J Clin Biochem 25: 208–12. 23105911 10.1007/s12291-010-0037-7 PMC3453101

[5] Marques-Vidal P, Bochud M, Bastardot F, Lüscher T, Ferrero F, et al (2012) Association between inflammatory and obesity markers in a Swiss population-based sample (CoLaus Study). Obes Facts 5: 734–44. 23108472 10.1159/000345045

[6] Zhu Y, Zhang Y, Ling W, Feng D, Wei X, et al (2011) Fruit consumptionis associated with lower carotid intima-media thicknessand C-reactive protein levels in patients with type 2 diabetesmellitus. J Am Diet Assoc 111: 1536–42. 21963020 10.1016/j.jada.2011.07.010

[7] Michigan A, Johnson TV, Master VA (2011) Review of the relationship between C-reactive protein and exercise. Mol Diagn Ther 15: 265–75. 22047154 10.1007/BF03256418

[8] Rojo-Martínez G, Soriguer F, Colomo N, Calle A, Goday A, et al (2013) Factors determining high-sensitivity C-reactive protein values inthe Spanish population. Di@bet.es study. Eur J Clin Invest 43: 1–10. 10.1111/eci.12002 23134526

[9] Belfki H, Ben Ali S, Bougatef S, Ben Ahmed D, Haddad N, et al (2012) Relationship of C-reactive protein with components of the metabolic syndrome in a Tunisian population. Eur J Intern Med 23: e5–9. 22153549 10.1016/j.ejim.2011.10.011

[10] Lenfant C, Chobanian AV, Jones DW (2003) Roccella EJ; Joint National Committee on the Prevention, Detection, Evaluation, and Treatment of High Blood Pressure (2003) Seventh report of the Joint National Committee on the Prevention, Detection, Evaluation, and Treatment of High Blood Pressure (JNC 7): resetting the hypertension sails. Hypertension 41: 1178–1179. 12756222 10.1161/01.HYP.0000075790.33892.AE

[11] Pearson TA, Mensah GA, Alexander RW, Anderson JL, Cannon RO 3rd, et al (2003) Markers of Inflammation and cardiovascular disease: application to clinical and Public Health practice: a statement for healthcare professionals from the centers for disease control and prevention and the American heart association. Circulation 107: 499–511. 12551878 10.1161/01.cir.0000052939.59093.45

[12] Wu S, Huang Z, Yang X, Zhou Y, Wang A, et al (2012) Prevalence of ideal cardiovascular health and its relationship with the 4-year cardiovascular events in a northern Chinese industrial city. Circ Cardiovasc Qual Outcomes. 5: 487–493. 10.1161/CIRCOUTCOMES.111.963694 22787064

[13] Wu S, Li Y, Jin C, Yang P, Li D, et al (2012) Intra-individual variability of high-sensitivity C-reactive protein in Chinese general population. Int J Cardiol 157: 75–79. 21215477 10.1016/j.ijcard.2010.12.019

[14] Mitchell JA, Bornstein DB, Sui X, Hooker SP, Church TS, et al (2010) The impact of combined health factors on cardiovascular disease mortality. Am Heart J 160: 102–108. 20598979 10.1016/j.ahj.2010.05.001 PMC2897813

[15] Odegaard AO, Koh WP, Gross MD, Yuan JM, Pereira MA (2011) Combined lifestyle factors and cardiovascular disease mortality in Chinese men and women: the Singapore Chinese health study. Circulation 124: 2847–2854. 22104554 10.1161/CIRCULATIONAHA.111.048843 PMC3400937

[16] Kvaavik E, Batty GD, Ursin G, Huxley R, Gale CR (2010) Influence of individual and combined health behaviors on total and cause-specific mortality in men and women: the United Kingdom Health and Lifestyle Survey. Arch Intern Med 170: 711–718. 20421558 10.1001/archinternmed.2010.76

[17] Nechuta SJ, Shu XO, Li HL, Yang G, Xiang YB, et al.. (2010) Combined impact of lifestyle-related factors on total and cause-specific mortality among Chinese women: prospective cohort study. PLoS Med 7: pii, e1000339. 10.1371/journal.pmed.1000339 PMC2939020 20856900

[18] Chuang SY, Hsu PF, Chang HY, Bai CH, Yeh WT, et al (2013) C-reactive Protein Predicts Systolic Blood Pressure and Pulse Pressure but not Diastolic Blood Pressure: the Cardiovascular Disease Risk Factors Two-Township Study. Am J Hypertens 26: 657–64. 23388833 10.1093/ajh/hps095

[19] Hung J, Knuiman MW, Divitini ML, Davis T, Beilby JP (2008) Prevalence and risk factor correlates of elevated C-reactive protein in an adult Australian population. Am J Cardiol 101: 193–198. 18178405 10.1016/j.amjcard.2007.07.061

[20] Labonté ME, Dewailly E, Chateau-Degat ML, Couture P, Lamarche B (2012) Population-based study of high plasma C-reactive protein concentrations among the Inuit of Nunavik. Int J Circumpolar Health 71. [Epub 2012 Oct 17]. 10.3402/ijch.v71i0.19066 PMC3475996 23087913

[21] Rabkin SW, Langer A, Ur E, Calciu CD, Leiter LA (2013) Inflammatory biomarkers CRP, MCP-1, serum amyloid alpha and interleukin-18 in patients with HTN and dyslipidemia: impact of diabetes mellitus on metabolic syndrome and the effect of statin therapy. Hypertens Res 7. [Epub ahead of print]. 10.1038/hr.2012.214 23388885

[22] Villegas R, Xiang YB, Cai H, Elasy T, Cai Q, et al (2012) Lifestyle determinants of C-reactive protein in middle-aged, urban Chinese men. Nutr Metab Cardiovasc Dis 22: 223–230. 21111583 10.1016/j.numecd.2010.07.007 PMC3143269

[23] WenJ, Liang Y, Wang F, Sun L, Guo Y, et al (2009) Association of C-reactive protein and metabolic syndrome in a rural Chinese population. Clin Biochem 42: 976–983. 19358836 10.1016/j.clinbiochem.2009.03.026

[24] Azadbakht L, Surkan PJ, Esmaillzadeh A, Willett WC (2011) The Dietary Approaches to Stop Hypertension Eating Plan Affects C-Reactive Protein, Coagulation Abnormalities, and Hepatic Function Tests among Type 2 Diabetic Patients. J Nutr 141: 1083–1088. 21525259 10.3945/jn.110.136739 PMC3137257

[25] Cozma A, Orăşan O, Sâmpelean D, Fodor A, Vlad C, et al (2009) Endothelial dysfunction in metabolic syndrome. Rom J Intern Med 47: 133–40. 20067163

[26] Kantor ED, Lampe JW, Kratz M, White E (2013) Lifestyle factors and inflammation: associations by body mass index. PLoS One 8: e67833. 23844105 10.1371/journal.pone.0067833 PMC3699492

[27] Hotamisligil GS, Shargill NS, Spiegelman BM (1993) Adipose expression of tumor necrosis factor-alpha: direct role in obesity linked insulin resistance. Science 259: 87–91. 7678183 10.1126/science.7678183

